# Preparation of Preformed Submicron Crosslinked Polymer Coils for Conformance Control in Low-Permeability Reservoirs

**DOI:** 10.3390/polym16010039

**Published:** 2023-12-21

**Authors:** Jianwei Liu, Bo Peng

**Affiliations:** Beijing Key Laboratory for Greenhouse Gas Storage and CO2-EOR, Unconventional Petroleum Research Institute, China University of Petroleum-Beijing, Beijing 102249, China; 2018310104@student.cup.edu.cn

**Keywords:** low-permeability reservoirs, conformance control, linked polymer coils, deep profile control, HPAM

## Abstract

With the increasing development of low-permeability reservoirs, the significance of conformance control treatment has risen considerably. To address the conflict between injectability and plugging performance, as well as to enhance the deep migration capacity of conformance control agents, preformed submicron crosslinked polymer coils (SCPCs) have been manufactured using aqueous solution dispersion polymerization. Fourier transform infrared and scanning electron microscopy were employed to examine the chemical structure and micromorphology, while particle size distribution, zeta potential, rheological, and filtration properties were analyzed. The effectiveness of conformance control was confirmed through the parallel core displacement. The effective particle size of SCPCs was at a submicron level (500~800 nm). SCPCs exhibit a transitional threshold concentration between gel and sol states (0.25 wt%~0.5 wt%). SCPCs can efficiently block the 1.2 μm microporous filter membrane. The filtration time is up to 67.8 min. SCPCs can improve the water absorption rate of lower permeability cores from 21.21% to 57.89% with a permeability difference of 5. Therefore, SCPCs have good injectability, plugging performance, and deep migration capacity and can be used for conformance control in low-permeability reservoirs.

## 1. Introduction

Low-permeability reservoirs are now the top priority for global oil companies due to the continuing depletion of conventional oil and gas resources [1]. Conformance control treatments are an effective means of enhancing economic benefits in low-permeability reservoirs, which are characterized by microfractures and high inhomogeneity [2]. However, meeting the demand for conformance control in low-permeability reservoirs using conventional proppants can be challenging due to the narrow pore size and complex pore structure of permeable reservoirs [3,4].

Polymer-based conformance control agents are currently the most widely used. These agents can be classified into two broad categories: in situ prepared agents and preformed agents. In situ prepared agents include colloidal dispersion gels (CDGs) [5,6], linked polymer solutions (LPSs) [7,8], etc. Preformed agents include dispersed particle gels (DPGs) [9,10,11], preformed particle gels (PPGs) [12,13], polymer microspheres (PMs) [14,15,16], etc.

Both CDGs and LPSs are dispersion systems of intramolecularly crosslinked polymer coils in water [17]. Typically, these systems are created by in situ crosslinking of polyacrylamide with an aluminum citrate crosslinking agent at low concentrations and then injecting mineralized water into the formation. The coils exhibit a strong plugging effect and significant deep migration capability. However, the in situ crosslinking reaction results in injection concentrations that are too low (≤1200 mg/L), and the formation is highly vulnerable to external conditions, such as the formation water.

DPGs, PPGs, and PMs are all preformed elastic gel particles that are then injected into the formation with water. This preformation process makes the injection concentration adjustable and less susceptible to the effects of formation temperature and mineralization. However, there is a conflict between the injectability and the plugging performance of the particles; the deep migration capacity is insufficient, and the particles are easily stuck or sheared in the wellbore, which seriously affects the effect of deep profile control.

These problems arise due to the distinct characteristics of coils and particles. Firstly, there is a difference in their blocking mechanism, wherein coils primarily rely on bridging, adsorption, retention, and similar effects to block the reservoir pore, whereas gel particles rely on the elastomer’s swelling to achieve blocking. Secondly, different crosslinking ratios result in varying deformability. The low crosslinked coils exhibit superior deformability, enabling easier pore deformation. Conversely, the highly crosslinked gel particles are excessively elastic, leading to entrapment within channels or shearing and breakage. Also, the coils in CDGs and LPSs measure between 200 and 800 nm following dissolution, making them submicrometer in scale and optimally suited for pore spaces in low-permeability reservoirs. Comparatively, the gel particles have a micrometer-level size post dissolution.

To resolve the conflict between the injectability and the blockability of the particles, He et al. [18] prepared controlled self-aggregating (CSA) particles by using styrene and acrylamide as monomers and N,N’-methylenebis-acrylamide as a crosslinking agent, and Li et al. [19] combined the CSA particles with surface activators, which had a good synergistic effect. The injection of CSA particles, which are significantly smaller than the target pore throat diameter, can not only successfully enter the deep porous medium, but also effectively block the high-permeability region by forming self-aggregating particle clusters. Zou et al. [20] synthesized self-assembled microspheres from DCA (divinylbenzene-co-acrylamide), and Shi et al. [21] conducted a subsequent investigation on the mechanism of plug formation and migration of DCA microspheres. These microspheres are capable of forming clusters in high-temperature and high-salinity oil–water environments. Remarkably, even when injected, DCA microspheres with diameters significantly smaller than the target pore throat could still effectively seal off the opening. This resulted in a tenfold increase in subsequent water drive pressure. Luo et al. [22] modified the reservoir through the grafting of cationic polymers on to the silica nanoparticles’ surface, resulting in hysteretic plugging and wetting inversion. This modification strengthened the particles’ plugging effect.

To solve the problem that microspheres are easily stuck in the channel or broken by shear, Yang et al. [23,24,25] prepared low-elastic polymer microspheres with good deformability by reducing the crosslinking ratio. Zhao et al. [26] prepared nanoscale polymer microspheres that can effectively penetrate into the deep part of low-permeability reservoirs and have a good plugging effect on the inhomogeneous core with the permeability of 10 μm^2^. Liu et al. [27] investigated the impacts of microsphere size, concentration and core permeability on plugging performance in highly permeable porous media with core displacement. Hou et al. [28] prepared submicron polymer microspheres and achieved good deep profile control effects in mine experiments in the Changqing oilfield.

The aforementioned research findings indicate that enhancing particle bridging and retention blocking, augmenting particle deformability, and reducing particle size to the submicron level can partially ameliorate the issues of injecting, blocking, and deep migrating in the process of drilling low-permeability reservoirs, but questions still remain on the improvement of particles, and researchers have not yet changed the nature of particle elastomers. The crosslinked polymer coils in CDGs and LPSs are a submicron-level system with strong deformability, bridging retention, and blocking orientation. This system is better suited for the demands of the deep migration in low-permeability reservoirs compared with particles, and it is only limited by the defects caused by in situ crosslinking, but it has not been widely used. The preparation of precrosslinked submicron polymer coils can offer a fresh approach to tackle challenges encountered in deep drive for low-permeability reservoirs.

Submicron cross-linked polymer coils (SCPCs) were synthesized via aqueous dispersion polymerization using aluminum citrate with a low crosslinking ratio, the preparation conditions were optimized, the chemical structure and micromorphology were characterized using Fourier transform infrared (FTIR) and scanning electron microscopy (SEM), and the particle size analysis was performed using dynamic light scattering. Based on this, the rheological and filtration properties of SCPCs were tested. The conditioning effect was verified through the double-tube–parallel-core flooding experiment. The research indicates that SCPCs share similar morphology with LPSs and possess excellent blocking performance, deep migration capability, and strong conformance control effects.

## 2. Materials and Methods

### 2.1. Material

Acrylamide (AM), acrylic acid (AA), ammonium sulfate, ammonium persulfate, sodium bisulfite, sodium hydroxide, sodium chloride, acetone, and ethyl alcohol, all analytically pure, were provided by Beijing Yili Fine Chemicals Company (Beijing, China). The dispersing stabilizer and aluminum citrate crosslinking agent were made in the laboratory.

### 2.2. Preparation of Coils

Weigh 7.5 g of AM and AA (molar ratio 7:3), 13 g of ammonium sulfate, 5 g of stabilizer, and 24.5 g of ionized water into a 500 mL four-neck–round-bottomed flask equipped with a condenser, thermometer, ammonia line, and stirrer, stir to dissolve, and adjust the pH to 4–6 with NaOH. Heat the mixture up to 30 °C. Vent nitrogen gas for 30 min, and then add ammonium persulfate and sodium bisulfite as redox initiators. After 22 h of reaction, the emulsion is obtained by adding a certain ratio of poly-aluminum and aluminum citrate crosslinking agent and warming at 40 °C for 10 h. Then wash the emulsion three times with anhydrous ethanol and acetone. After multiple centrifugations, dry the emulsion in an oven at 40 °C for 24 h to yield the powder sample of coils.

### 2.3. Characterization

The chemical structure of the coils was analyzed using the WQF-520 FTIR spectrometer (Beijing Beifang Rayleigh Analytical Instruments (Group) Co., Ltd., Beijing, China). The KBr compression method was employed with a scanning range between 600 and 4000 cm^−1^ and a scanning time of 24 scans. The resolution was less than 2 cm^−1^. The samples were dissolved in deionized water as a 10 mg/L solution, then applied to the conductive adhesive and dried in the shade for 12 h. The samples were analyzed utilizing a JSM-8010 cold-field emission SEM (Hitachi, Ltd., Tokyo, Japan) to observe their microscopic morphology.

### 2.4. Particle Size Distribution and Zeta Potential Measurements

A Delsa TM Nano nanoparticle size and zeta Potential analyzer (Beckman Coulter, Inc., Pasadena, CA, USA) was utilized to ascertain the coils’ efficient size, size distribution, and zeta potential. The polymer coils were diluted in a solution (1000 mg/L), dispersed through ultrasonication, and placed in a cuvette preheated for 30 min at a temperature of 25 °C and a scattering angle of 15°. The process was repeated thrice for each sample to acquire the effective size and size distribution. After cuvette replacement, the zeta potential of the samples was measured. Configuring the polymer coil powder that had been dried as a 0.1 wt% solution in a stoppered reagent bottle, then subjecting it to various solubilization conditions and measuring the effective size of the samples over a period of seven days was performed in order to evaluate the solubility and time stability of the polymer coils [29].

### 2.5. Rheology Measurements

For rheological characterization, steady state shear and dynamic oscillatory shear rheological tests were performed using a Haake RS600 rheometer (Thermo Fisher Scientific, Waltham, MA, USA) and a coaxial cylindrical sensor system (Z41Ti). The temperature was controlled at 30 °C ± 0.05 °C with the DC 50 temperature controller (Thermo Fisher Scientific, Waltham, MA, USA). In the steady shear test, the shear rate was gradually increased from 0.001 s^−1^ to 1000 s^−1^ with a gradient of less than 0.5% (∆τ/τ)/∆t, and the maximum waiting time was 30 s. Different samples were prepared and dissolved for 12 h at 30 °C. During the oscillatory shear test, the scanning frequency was kept fixed at 1 Hz, and stress scanning was used to identify a linear viscoelastic region, and then the frequency (ω) 0.5 (∆τ/τ)/∆t% was measured after selecting the appropriate stress level. The energy storage modulus (G′) and loss modulus (G″) were both measured over frequencies (ω) ranging from 0.01 Hz to 100 Hz.

### 2.6. Microporous Membrane Filtration Experiment

**Figure 1:**
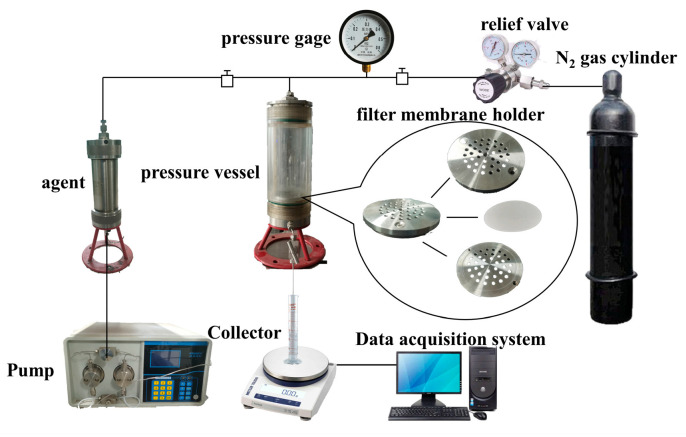
Schematic of the microporous membrane filtration experiment.

The blocking properties of coils were investigated through microporous filtration membrane experiments, as depicted in Figure 1. Solutions were prepared as different samples and dissolved for 12 h at 30 °C. The selected membranes were placed flat in a membrane holder and moistened with deionized water. The samples to be tested were placed in a piston flask. A stable pressure of 0.05 MPa was applied via the nitrogen cylinder using a pressure reducing valve. The solution to be tested was then injected through an advection pump. After allowing it to stand for 3 min to observe any air leakage, the amount of filtrate passing through the membrane and the filtration time were recorded using an electronic balance and computer recording software(Provided by Jiangsu Tuochuang Scientific Research Instrument Co., Ltd., Hai’an City, Jiangsu Province, China).

### 2.7. Core Flooding

**Figure 2:**
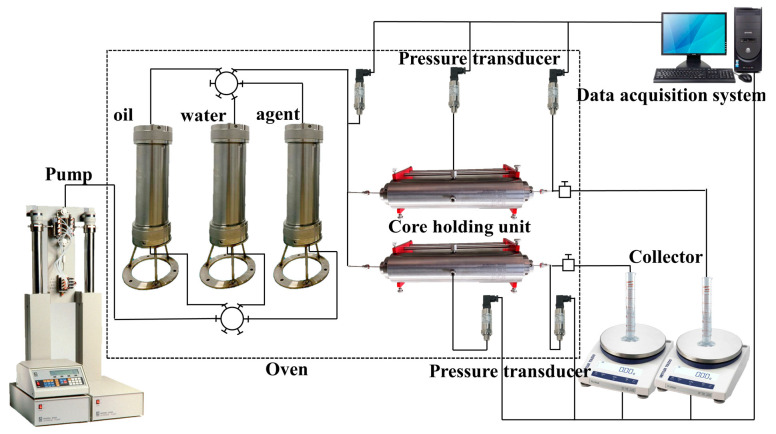
Schematic of the parallel core displacement.

At a temperature of 80 °C, a parallel core exchange experiment was conducted using parallel cores with a permeability ratio of 1:5. Our objective was to investigate the effects of coil conditioning as shown in Figure 2. First, the permeability of the core samples was measured with gas. According to Table 1, the composition of the formation water is presented. The simulated formation water was formulated based on the ionic composition of the formation water in the Daqing Lamaidean reservoir [30]. The core was saturated with formation water for 10 h at room temperature. After that, it was saturated with oil by using the core gripper at 20 MPa and 80 °C for 24 h. The volume of the saturated oil was calculated by measuring the volume of formation water in the extracted fluid. Following this, the driving experiment began. The pertinent core-related parameters are displayed in Table 2. The trial injection rate was established at 0.3 mL/min at a temperature of 80 °C. Subsequently, water injection, equaling 1 pore volume, was executed. After injecting a 0.1% wt solution of groundwater from the coils (1.5 pore volume). Finally, postinjection of water was performed. Absorption rates of high- and low-permeability cores at each stage, along with pressure changes before and after the gripper, were measured [31].

## 3. Results and Discussion

### 3.1. Characterizations of SCPCs

#### 3.1.1. Chemical Structure of Polymer Coils

**Figure 3:**
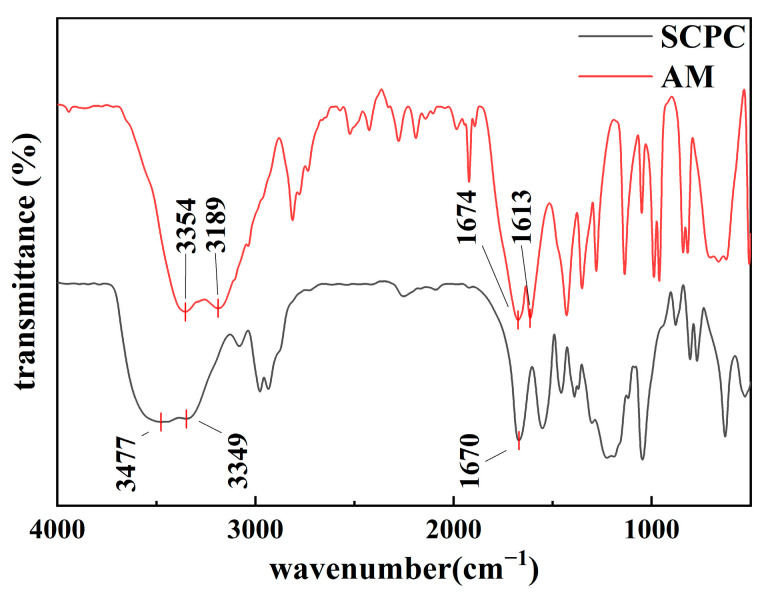
Fourier transform infrared spectra of acrylamide and submicron crosslinked polymer coils (SCPCs).

The FTIR spectra of AM and SCPCs are presented in Figure 3. The absorption peak at 3477 cm^−1^ in the FTIR spectrum of SCPCs corresponds to the stretching vibration peak of -OH, which is the distinguishing absorption peak of AA. Compared to the spectrum of AM, the N-H absorption peak at 3349 cm^−1^ is masked by the stretching vibration peak of -OH. However, the C=O stretching vibration peak in the amide group at 1670 cm^−1^ still exists, while the C=C stretching vibration peak at 1613 cm^−1^ disappears, which is the distinguishing absorption peak of AM. This suggests that the polymerization reaction successfully involved both AM and AA.

#### 3.1.2. Morphology of Polymer Coils

**Figure 4:**
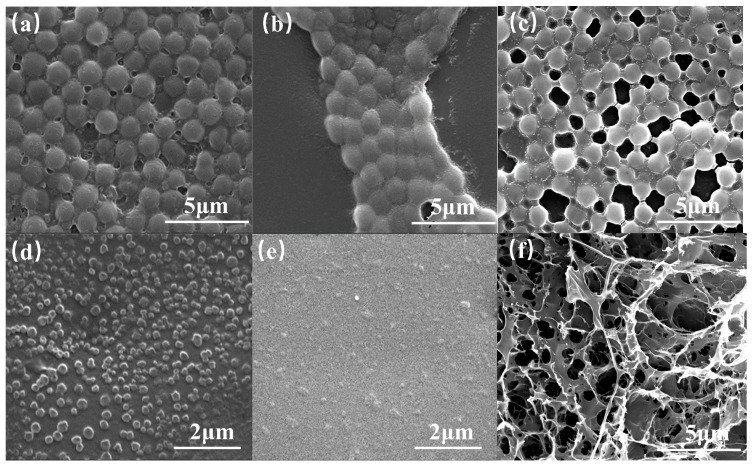
Scanning electron microscopy photos of 0.01wt% polymer coils with different crosslinking ratios (**a**) 2%; (**b**) 1%; (**c**) 0.5%; (**d**) 0.25%; and (**e**) 0%. (**f**) Scanning electron microscopy photos of 1%wt polymer coils with a crosslinking ratio of 0.25%.

Micrographs of the shade-dried polymer coils with various crosslinking ratios of 10 mg/L are depicted in Figure 4. As the crosslinking ratio decreases, the size of the shade-dried polymer coils gradually reduces, and the morphology becomes irregular. As the crosslinking ratio decreases, the coils in the structure gradually reduce their three-dimensional network, leading to diminished bound-water levels and increased deformability. Consequently, extrusion and drawing can easily alter the coils’ shape.

It should be noted that the coils have a significant reticular connection at the crosslinking ratio of 0.5% and experience a notable decrease in size when the crosslinking ratio is reduced to 0.25%. This can be attributed to the dispersion polymerization synthesis mechanism.

**Figure 5:**
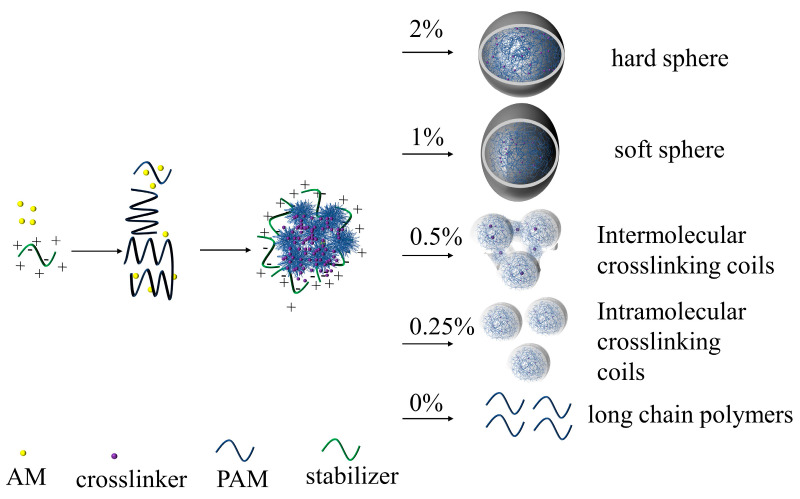
Polymer coils at different crosslinking ratios.

During dispersion polymerization, emulsion droplets can contain multiple polymer molecules. These droplets stabilize through charge, space-site resistance, hydrogen bonding, and crosslinking sites [32]. When the powdered coils were dissolved in deionized water to form a dilute solution of 10 mg/L, the charge, space-site resistance, and hydrogen bonding exhibited significant weakening. Furthermore, the morphology was solely limited by the crosslinking sites. As shown in Figure 5.

When the crosslinking ratio is 0%, the polymers in the droplets are hydrogen bonded, so when a dilute solution is made, the hydrogen bonds are completely opened to long chain polymers. Due to the lower concentration and molecular weight, this phenomenon cannot be observed with SEM. As shown in Figure 4e.

**Figure 6:**
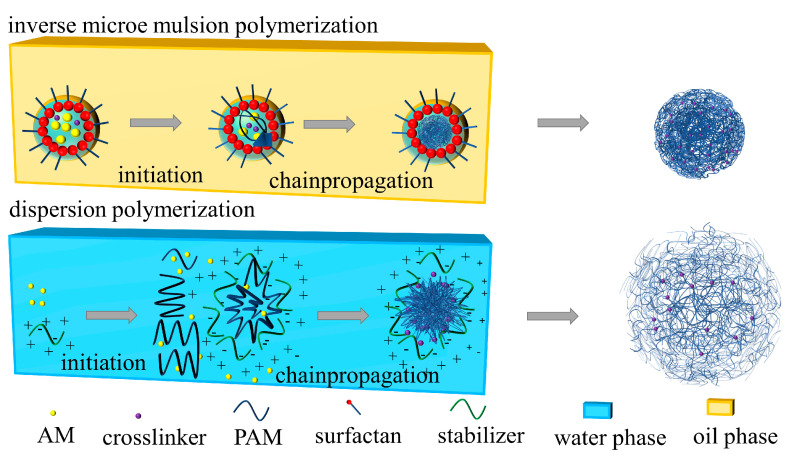
Differences between dispersion polymerization and microemulsion polymerization.

When crosslinking is at a level of 0.25%, the size of intramolecular crosslinked coils is significantly smaller than the emulsion diameter, as illustrated in Figure 4d. This is different from the phenomenon of Wang et al. [32]. This difference arises due to the distinct nature of dispersion polymerization as opposed to emulsion polymerization. In the case of aqueous solution dispersion polymerization, the dispersion medium comprises a high concentration of electrolyte, which is demonstrated in Figure 6. As the electrolyte concentration increases, the polymer molecules curl and shrink, leading to more intramolecular crosslinking reactions. Consequently, intramolecular crosslinking is the preferred outcome in dispersion polymerization.

When the crosslinking ratio reaches 0.5%, intermolecular crosslinking occurs, resulting in the formation of a distinct mesh structure between the coils, as illustrated in Figure 4c.

When the crosslinking ratio is 1%, the number of crosslinking sites increases, allowing the coils in the emulsion droplet to form a complete mesh structure. However, due to a low number of crosslinking sites, they are prone to squeezing and deformation into ellipsoidal or polygonal shapes, producing a “soft ball” state, as depicted in Figure 4b.

When the crosslinking ratio is set at 2%, the crosslinking sites continue to increase, causing the coils to gelatinize and form a spherical shape. This formation is manifested as a “hard ball,” as shown in the Figure 4a.

Due to the crosslinking ratio of 0.25%, the coils exhibited distinct size, morphology, and significantly improved deformability compared to other crosslinking ratios. Therefore, they were designated as SCPC for the purposes of this study.

The SEM results for the shade-dried 1%wt SCPCs are displayed in Figure 4f, revealing coils with a distinct reticular structure. This structure arises from the SCPCs’ robust intermolecular interaction capacity that enables them to undergo a gel–sol phase change, developing a composition similar to an overall gel at higher concentrations that exhibits unique traits in contrast to individual particles.

### 3.2. Particle Size and Zeta Potential Analysis

**Figure 7:**
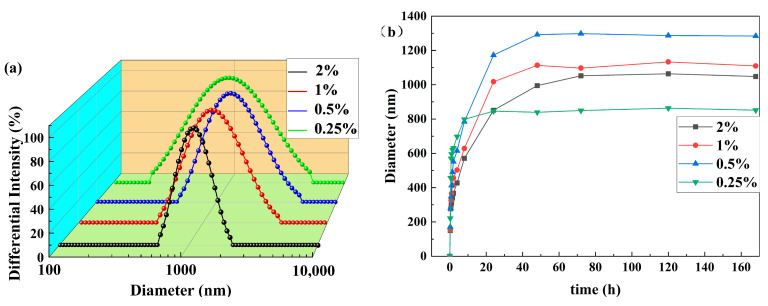
Effect of crosslinking ratio on coils (**a**) distribution and (**b**) effective size.

The effective sizes of the crosslinked coils with various crosslinking ratios are presented in Figure 7a. The results demonstrate that size and size distribution are larger and wider when the crosslinking ratio is between 0.5% and 2%. When the coils are dispersed in an aqueous solution, –CONH_2_ is hydrolyzed to –COOH. Electrostatic repulsion between the neighboring carboxyl groups causes the long chains in the coils to stretch. When the crosslinking ratio decreases, the number of crosslinking sites decreases, causing the polymer chains to stretch further. As a result, the deformability and intermolecular interactions become stronger, leading to an increase in size and a wider sizes distribution. When the crosslinking ratio reaches 0.25%, the size of the coils decreases significantly, and the size distribution becomes wider. This occurs because if the crosslinking ratio is too low, only aluminum citrate’s intramolecular crosslinking takes place within the coils. After dilution, this breaks down into polymer coils of a submicron level. These findings align with the consistent results of Figure 4.

The dissolution curves for the polymer coils are presented in Figure 7b, illustrating that with a decreasing crosslinking ratio, the time required for maximum dissolution decreases gradually. There is no significant alteration observed in the size during the following 7 days. The swelling time is shortened as the number of crosslink sites is reduced, which makes it easier for water molecules to penetrate the three-dimensional network structure.

Unlike particles with a concentrated size distribution and slower dissolution rate, SCPCs exhibit stronger deformability and intermolecular interactions. They exhibit wider size distribution and a faster dissolution rate. It can be inferred that SCPCs boast strong solubility, deformability, retention blocking ability, and bridging blocking ability.

**Figure 8:**
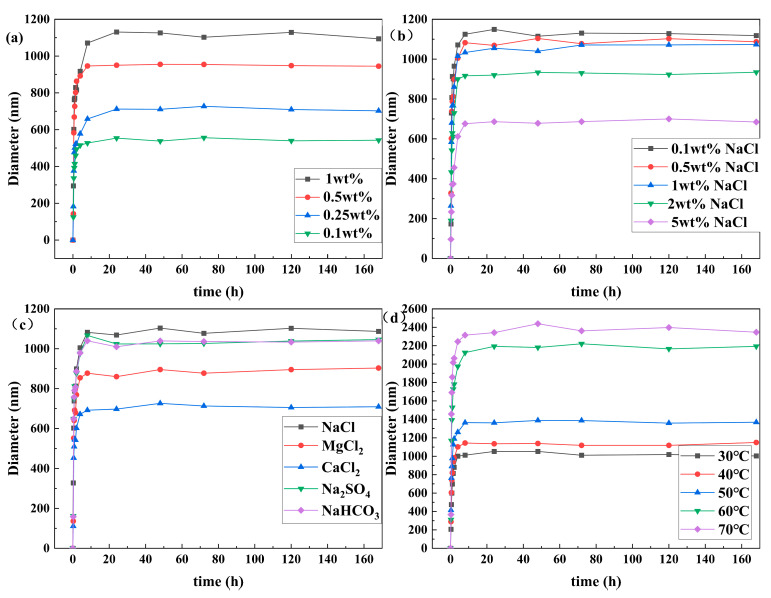
Effect of different conditions on polymer coils size (**a**) concentration, (**b**) NaCl concentration, (**c**) ionic species, and (**d**) temperature.

The influence of concentration on the size of SCPCs is displayed in Figure 8a. As the concentration increases, the average size of SCPCs progressively grows. The stronger intermolecular connections of polymer coils compared to particles enhance the bridging effect between them, producing a significant rise in the average size of the polymer coils. The size increase was greater at a concentration gradient of 0.25% to 0.5%. This aligns with the critical concentration determined from subsequent rheological assessments.

The impact of NaCl concentration on SCPC size is illustrated in Figure 8b. Increasing NaCl concentration results in a smaller average size of the polymer coils. This occurs because higher NaCl concentration leads to an increase in Na^+^ content, which enters the interior of the polymer coils. Consequently, the difference between the internal and external ion concentration creates osmotic pressure that compresses the double electric layer and reduces the average size.

The impact of ionic species on the size of SCPCs is illustrated in Figure 8c. The influence of Ca^2+^ and Mg^2+^ ions on the average size of polymer coils exceeded that of Na^+^, SO_4_^2−^, and HCO_3_^2−^. In addition, their dissolution rate was significantly lower than that of the latter. When the polymer coils are dissolved in water, the amide group undergoes hydrolysis to form a carboxyl group, resulting in the formation of an anionic polymer. As a result, cations are distributed around the anionic group, creating a stable electric field. When external cations are present, they shield the negative charge on the polymer anion, resulting in weakened electrostatic repulsion among the polymer molecules. The positive charge density of Ca^2+^ and Mg^2+^ in aqueous solutions is higher than that of Na^+^, leading to stronger shielding ability and a smaller size of the polymer coils.

The impact of temperature on the size of SCPCs is demonstrated in Figure 8d. The average size of polymer coils at the same temperature grows with an increase in dissolution time. The average size under different temperatures rises with increasing temperature. At 60 °C, the polymer coils’ size increases significantly. The increase in temperature intensifies the hydrolysis of amide groups and other groups in the polymer coils, leading to an increase in the degree of hydrolysis in these groups. This, in turn, enhances the water-absorbing and swelling properties, resulting in a larger particle size at high temperatures compared to low temperatures. The influence of synthesis conditions on the particle size of SCPCs is shown in Figures S3 and S4.

Under the selected NaCl concentration, ionic species, and temperature conditions, the size of SCPCs varied but remained stable over seven days. This confirms the excellent salt and temperature resistance of SCPCs.

**Figure 9:**
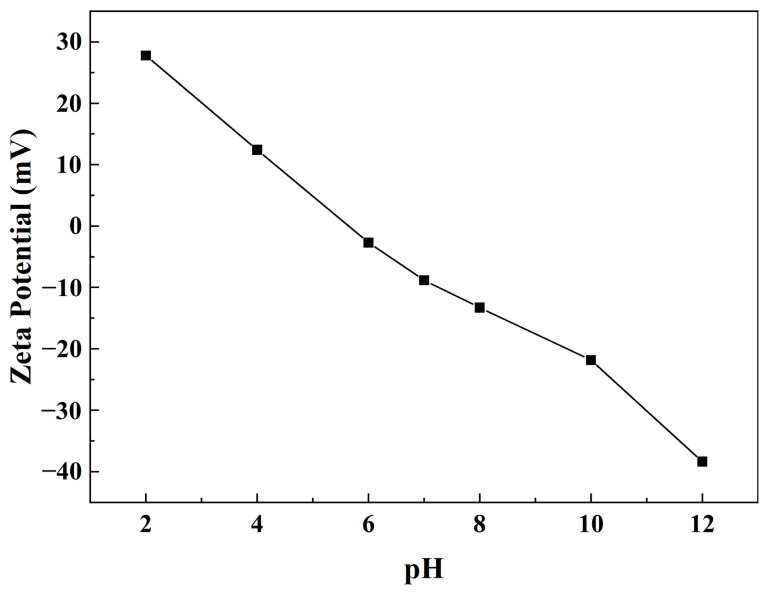
Zeta potential of SCPCs at different pH levels.

The zeta potentials of SCPCs at various pH levels are displayed in Figure 9. As the pH rises from 2 to 12, the zeta potential reduces from positive to negative values, and the isoelectric point is between pH 4 and 6. This suggests that the polymer coils are anionic polyacrylamides, in which the acrylic monomer can be hydrolyzed to carboxyl groups. As the pH decreases, the concentration of H^+^ ions gradually increases. This results in the neutralization of the negative charge carried by the carboxyl group, which then tends to carry a more positive charge. At the chosen pH, except for near the isoelectric point (pH 4–6), the absolute values are greater than 10 mV. Consequently, the SCPC colloid exhibits relatively stable properties.

### 3.3. Rheology

**Figure 10:**
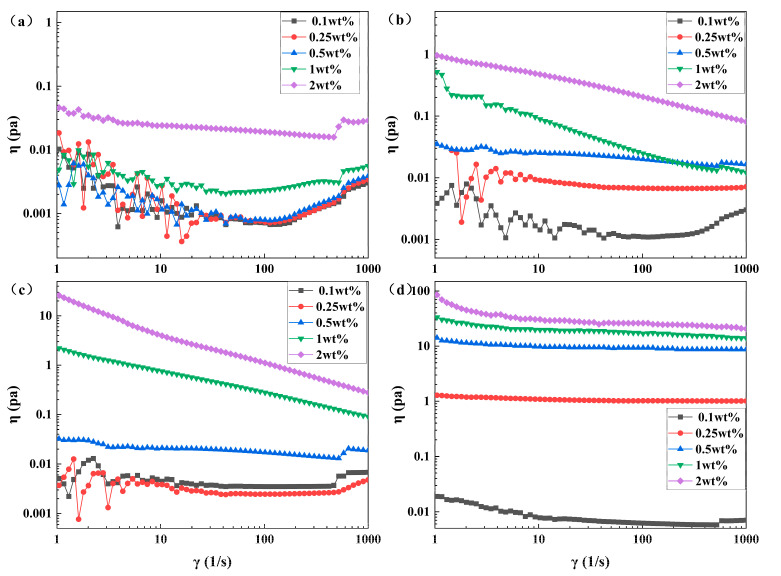
Steady state shear curves of polymer coils with different crosslinking ratios of (**a**) 2%, (**b**) 1%, (**c**) 0.5%, and (**d**) 0.25% for different concentrations of polymer coils.

From Figure 10, it is evident that η increases gradually with a decrease in crosslinking ratio at a specific concentration, and η decreases gradually with a decrease in concentration at a certain crosslinking ratio.

Figure 10a illustrates how the viscosity (η) changes with changing shear rate ($\dot{\gamma }$) for different mass fractions of polymer coils in solution at a crosslinking ratio of 2%. The fluid displays a Newtonian plateau for various mass fractions, becomes pseudoplastic at low shear rates, shows approximately Newtonian behavior at intermediate shear rates, and behaves as an expanding fluid at high shear rates.

When the crosslinking ratio is 0.5% or 1%, Figure 10b,c demonstrate the alteration of η with $\dot{\gamma }$ for various mass fractions of polymer coils in the solution. For mass fractions ω ≤ 0.5%, the Newtonian plateau becomes more pronounced with an increase in mass fraction. Conversely, when the mass fraction ω > 0.5%, the Newtonian plateau vanishes, η reduces with an increase in $\dot{\gamma }$, and the solution transforms into a pseudoplastic fluid.

When the crosslinking ratio is 0.25%, Figure 10d illustrates the variation of η with $\dot{\gamma }$ for different mass fractions of polymer coil solutions. For a mass fraction of ω = 0.1, the results are displayed. At a shear rate of 1 s^−1^ to 60 s^−1^, the solution exhibits pseudoplastic fluid behavior. At a shear rate of 60 s^−1^ to 313 s^−1^, the solution demonstrates approximations to a Newtonian fluid with a Newtonian plateau. When the shear rate is between 313 s^−1^ and 1000 s^−1^, the Newtonian plateau disappears, and the curves illustrating the change in η with $\dot{\gamma }$ exhibit an increase as $\dot{\gamma }$ increases, resulting in an expanding fluid. When the mass fraction exceeds 0.25%, the Newtonian plateau disappears. As $\dot{\gamma }$ increases, the curve of η versus $\dot{\gamma }$ decreases, and the solution becomes a pseudoplastic fluid. The more the mass fraction increases, the more apparent the shear thinning behavior becomes.

The experimental results above demonstrate that the rheological characteristics of polymer coils with varying concentration levels exhibit significant differences. In diluted solutions, the coils exist independently of one another. As the coil concentration increases, aggregation occurs, resulting in the appearance of a Newtonian platform. The higher the coil concentration, the more visible the platform. At high coil concentrations, the agglomerated coils are destroyed by the shear stress, which leads to shear thinning behavior. As the ratio of crosslinking decreases, the critical concentration needed for the Newtonian platform to appear decreases as well.

**Figure 11:**
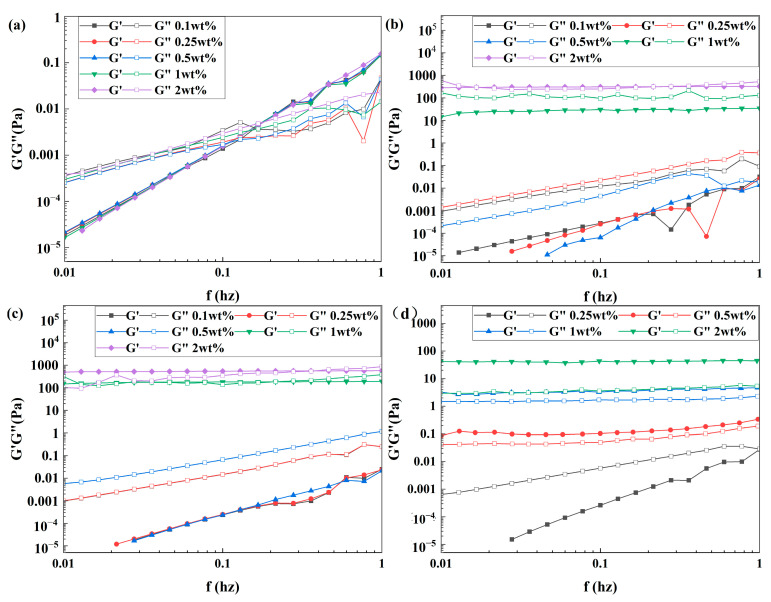
Dynamic oscillation curves with different crosslinking ratios of (**a**) 2%, (**b**) 1%, (**c**) 0.5%, and (**d**) 0.25% for different concentrations of coils.

The graphs in Figure 11a depict the changes in G′ and G″ when varying the mass fractions of nematic solutions at a crosslinking ratio of 2%. The coils consistently exhibit a lower G′ than G″ at low frequencies, where the dominant response is viscous. At mid-frequencies, the two moduli intersect, and at high frequencies, the elastic response dominates where G″ remains lower than G′. The coils possess clear characteristics of concentrated solutions or entangled networks, and there is no correlation between G′ and G″ and the concentration within the selected frequency range. The increase in viscoelasticity of the dispersed solution is due to the three-dimensional mesh structure within the coils resulting from a crosslinking ratio of 2%. This structure prevents water molecules from entering the coils, thereby promoting better dispersion in the solution. Furthermore, the reduced likelihood of intermolecular interactions between the polymer coils is consistent with the results of the steady-state shear curve.

The variations of G′ and G″ for different mass fractions of coil solutions with crosslinking ratios of 0.5% or 1% are displayed in Figure 11b,c. At low frequencies, G′ is consistently lower than G″ when ω ≤ 0.5%, indicating a dominant viscous response. At high frequencies, the two moduli intersect, which indicates that the solutions are concentrated or entangled networks of coils at this point. The frequency-dependent behavior of G′ and G″ can be attributed to the lower frequency’s tendency to result in coil deformation, leading to a viscous response over time. Conversely, at higher frequencies, molecular rearrangement takes longer than the oscillation period, resulting in temporary entanglement and the formation of a short-lived–three-dimensional network, which allows G′ to predominate. When the concentration of polymer coils reaches 1%, G′ always dominates G″ and demonstrates solid-like behavior, meaning deformation within the linear range is essentially recoverable. This suggests that as concentration increases, the coils transition into a sol–gel state, forming a weak gel. The critical concentration for the transition is predicted to be between 0.5% and 1%, in line with the results of the steady-state shear curve.

Figure 11d illustrates the alterations in both G′ and G″ regarding different mass fractions of coil solutions when the crosslinking ratio is 0.25%. At ω = 0.25%, G″ exceeds G′ at a lower frequency indicating a more prominent viscous response. The meeting point of G′ and G″ at higher frequency signifies the formation of concentrated solutions or tangled networks by the coils. When the coil concentration reaches 0.25% or higher, G′ becomes parallel to G″ and consistently dominates, suggesting that increasing the concentration leads to a sol–gel state transition due to the good deformability of the coils. The predicted critical concentration for the gel to sol transition falls between 0.25% and 0.5%, which agrees with the results of the steady-state shear curve.

Figure 11 demonstrates a reduction in the three-dimensional mesh structure of the coils alongside a gradual increase in deformability and enhancement of intermolecular interactions with a decreasing crosslinking ratio. At concentrations above the critical level, a sol–gel state transition occurs. Additionally, the critical concentration decreases as the crosslinking ratio decreases. This unique concentration dependence in rheology differs from that of the particles and aligns with the steady-state shear findings.

### 3.4. Filtration Curves

**Figure 12:**
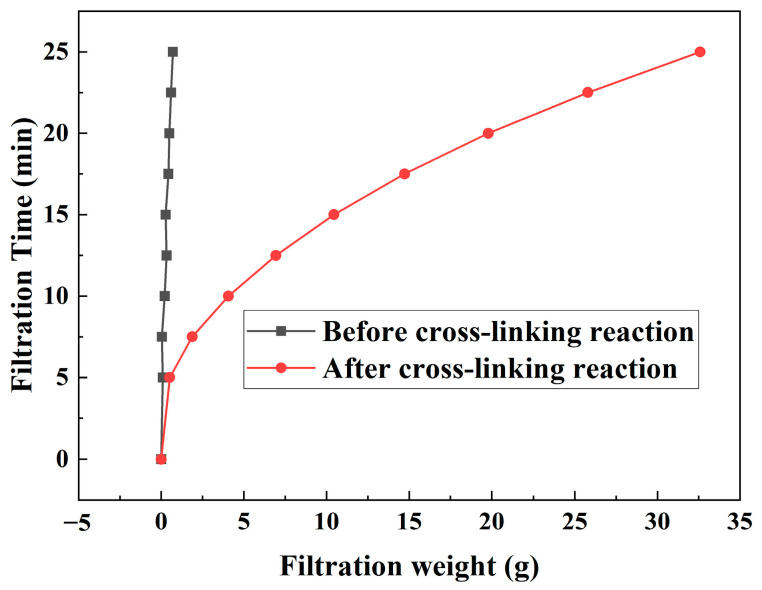
Effect of crosslinking reaction (crosslinking ratio 0.25%) on the filtration curve of polymer coils (0.25 wt%).

The filtration curves of the coils before and after crosslinking are presented in Figure 12. The filtration behaviors differ significantly. Prior to crosslinking, the filtration curve is a straight line, maintaining a constant filtration rate, and does not clog the membrane. Post crosslinking, the filtration curve takes on a parabolic shape, with a gradual and slow decrease in the filtration rate, effectively blocking the 1.2 μm microporous membrane. The above results demonstrate that the precrosslinked coils, although clustered under the influence of external electrolytes, can extend into long-chain polymers that pass through the filter membrane quickly without obstruction. Post crosslinking, the morphology of the coils is restricted by the presence of crosslinking points that can cause bridging blockages within the filter membrane pores, leading to superior blocking performance.

**Figure 13:**
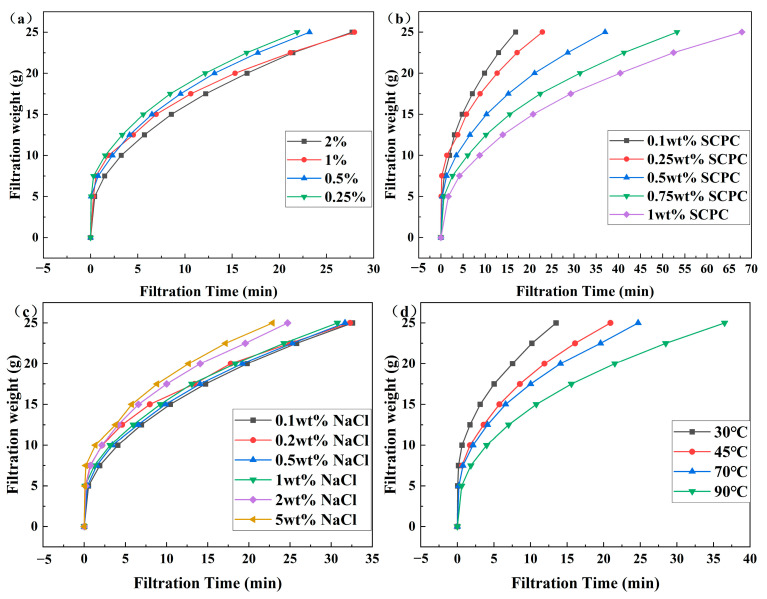
Effect of different conditions on the filtration curve of polymer coils (**a**) crosslinking ratio, (**b**) SCPC concentration, (**c**) NaCl concentration, and (**d**) temperature.

**Figure 14:**
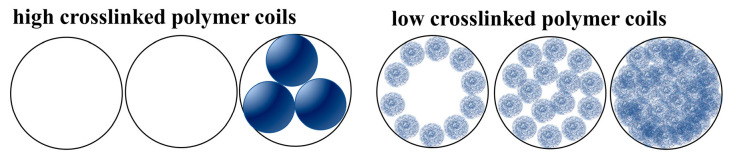
Difference in blocking mechanism of high/low crosslinked coils.

The effect of the crosslinking ratio on the filtration curve is shown in Figure 13a. The ability to block weakens as the ratio decreases, but the difference is not significant. As the crosslinking ratio decreases, the three-dimensional mesh structure inside the coils also decreases, which affects the blocking effect of the polymer coils. However, as shown in Figure 7a,b, if the crosslinking ratio is greater than 0.25%, as the crosslinking ratio decreases, the particle size of the coils increases, which strengthen the blocking effect of the polymer coils. Therefore, the impact of the blocking effect is slight. Nevertheless, if the crosslinking ratio falls below 0.25%, the average particle size of the coils notably decreases. However, the SCPCs’ strong intermolecular interactions, together with a concentration exceeding their critical point, can cause adsorption retention and bridging blockage, leading to a transfer to the gel state from the sol state. Consequently, an efficient filter membrane blocking can be achieved. The difference in blocking mechanism is shown in Figure 14.

The impact of concentration on the filtration curve of SCPCs is depicted in Figure 13b, revealing that as the concentration increases, the blocking effect intensifies. Greater concentration leads to a higher number of coils per unit of volume, an augmented adsorption retention, bridging blocking, and a greater blocking rate of the microporous filtration membrane, all of which contribute to an intensified blocking effect. Additionally, based on the DLS findings presented in Figure 8a, an increase in concentration results in an increase in particle size of SCPCs, which further enhances the blocking effect.

The impact of NaCl concentration on the filtration curve of SCPCs is illustrated in Figure 13c. Increasing the concentration of NaCl results in a decrease in blocking performance, with a more pronounced effect observed for salt concentrations above 2%. Figure 8b displays that an increase in NaCl mass concentration causes a reduction in SCPC particle size, which in turn affects the blocking effect.

The impact of temperature on the filtration curve of SCPCs is depicted in Figure 13d and illustrates a decline in blocking performance with rising temperature. This is attributable to the degradation of crosslinked polymer molecules under high temperature and oxygen conditions, leading to the breaking of crosslinked molecular chains. Additionally, Figure 8d illustrates that the particle size of SCPCs decreases as temperature increases, which ultimately results in a reduction in blocking capacity. Influence of stabilizer intrinsic viscosity and aging time on filtration performance were shown in Figures S1 and S2.

### 3.5. Displacement 

**Figure 15:**
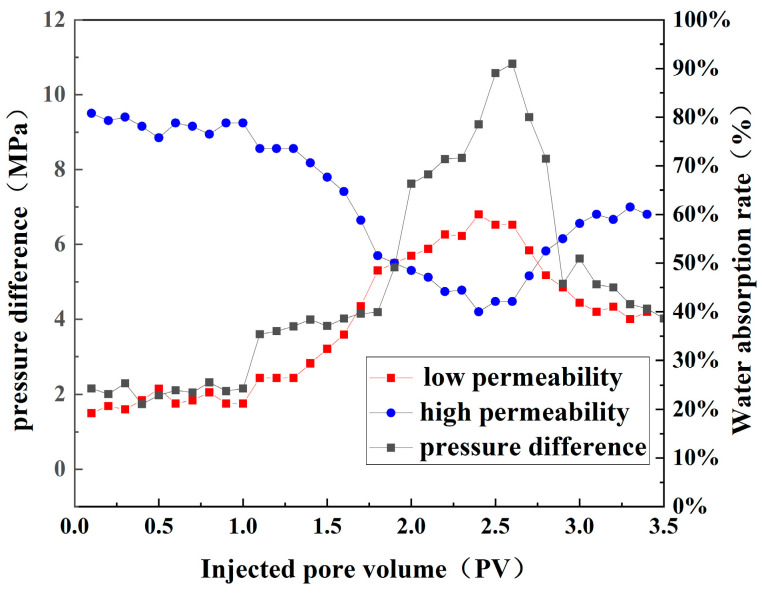
Pressure difference between front and back end and split flow rate in high- and low-permeability cores in double-pipe–parallel-drive alternation.

As indicated in Figure 15, during the water flooding stage, the low-permeability core exhibits a water absorption rate of less than 20%, which demonstrates the significant impact of nonhomogeneity on wave efficacy. Upon the injection of SCPCs, there was a marked increase in pressure differential between the front and back ends, resulting in a significant rise in shunt flow within the low-permeability core. SCPCs can temporarily block the pore throat in the high-permeability core, redirect fluid flow deeper, and greatly increase the shunt flow in the low-permeability core. As a result, in the subsequent water injection process, the driving pressure significantly decreases and the shunt flow rate in the low-permeability core also decreases significantly. Most of the SCPCs entered the deeper part of the formation during subsequent water injection in the post water drive process. As a result, SCPCs demonstrate strong plugging performance and deep transportation ability and have a more effective conformance control.

## 4. Conclusions

Addressing the issue of contradiction between injection performance and blocking performance of conformance control agents in low-permeability reservoirs and insufficient deep migration capacity, this research prepared preformed SCPCs via dispersion polymerization. The SEM analysis showed high deformability of SCPCs, and the microscopic morphology was similar to that of crosslinked polymer coils in CDGs and LPSs. Particle size analysis indicated that the effective particle size of SCPCs was at a submicron level, enabling it to dissolve rapidly and thoroughly, and maintain particle size stability for seven days, while exhibiting resistance to certain levels of temperature and salt. Rheological experiments revealed that SCPCs exhibit robust intermolecular interactions, and the transitional threshold concentration between gel and sol states ranges from 0.25% to 0.5%. This transition occurs effortlessly at low concentrations, which aids in blocking channels via retention enrichment or bridging. Subsequent experiments on the filter membrane and core replacement show that SCPCs possess considerable deformability and plugging performance. Thus, it can efficiently block the 1.2 μm microporous filter membrane through retention and bridge blocking and manage flow reversal for low-permeability cores with a permeability polarity difference of 5. This leads to a positive effect on deep drive regulation.

## Figures and Tables

**Table 1:** The simulated formation water ionic composition.

Ion Species	K^+^+Na^+^	Ca^2+^	Mg^2+^	SO_4_^2−^	HCO^3−^	CO_3_^2−^	Cl^−^	Salinity
Content mg/L	2428.01	14.85	7.48	54.1	2160.08	197.66	2266.88	7156.5

**Table 2:** Core parameters.

Core	Length (cm)	Diameter (cm)	Gas Permeability (mD)	Water Permeability (mD)	Pore Volume (mL)	Porosity (%)	Saturated Oil (mL)
low permeability	10.009	3.792	21	11.9	18.54	16.47	8.2
high permeability	9.973	3.792	95	34.0	17.44	15.49	15.8

## Data Availability

Data will be made available upon request.

## Supplementary Materials

The following supporting information can be downloaded at: https://www.mdpi.com/article/10.3390/polym16010039/s1, Figure S1: Influence of aging time on filtration performance; Figure S2: Influence of stabilizer intrinsic viscosity on filtration performance; Figure S3: Influence of stabilizer molecular weight (A1 > A2 > A3) in coils size on the reaction; Figure S4: Influence of stirring speed on coils size in the reaction.
