# Supplementary figures and images for: Preparation of Preformed Submicron Crosslinked Polymer Coils for Conformance Control in Low-Permeability Reservoirs
Source: Polymers (Basel). 2023 Dec 21;16(1):39. doi: 10.3390/polym16010039 (PMC10780422; doi:10.3390/polym16010039)

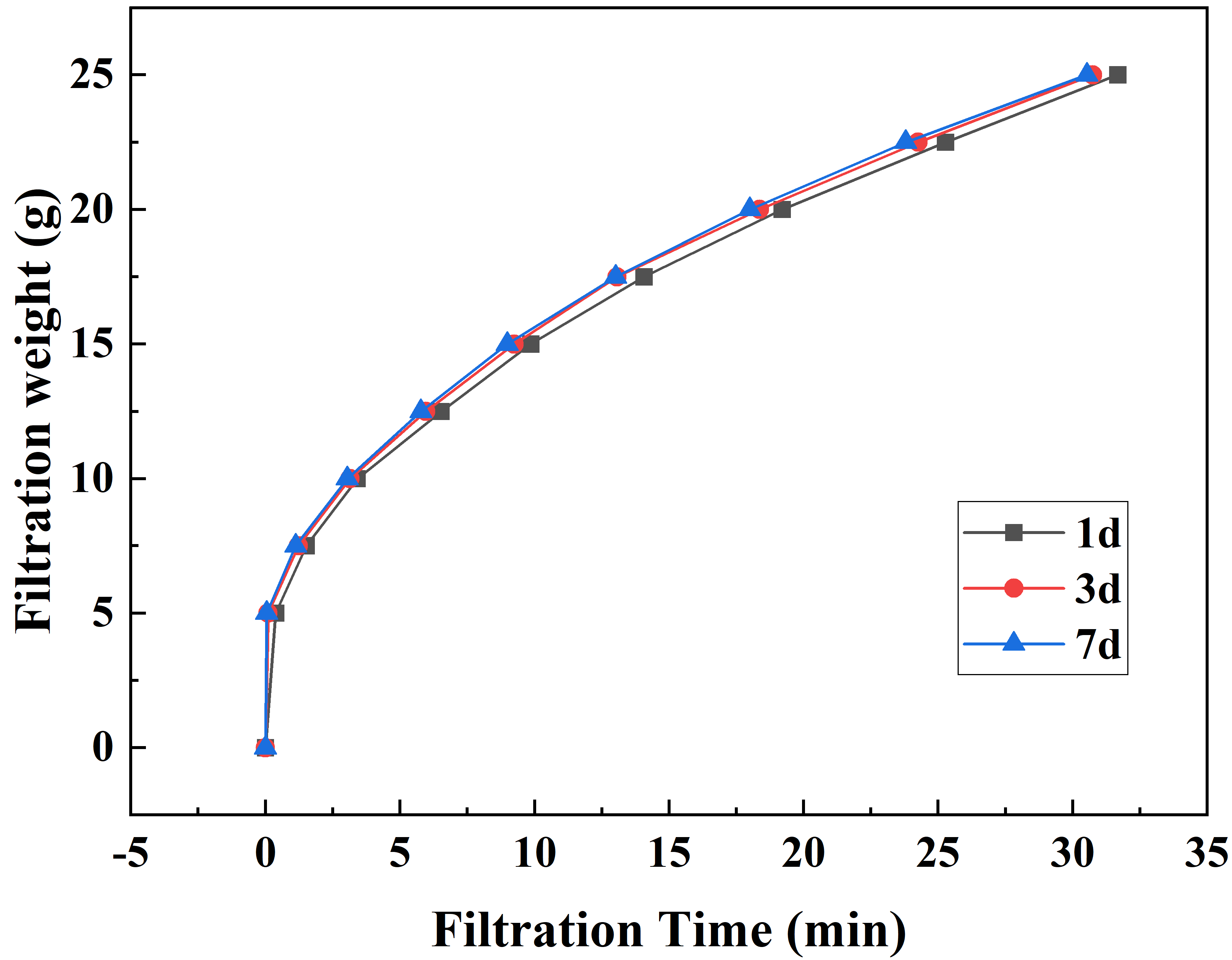

Supplement: Supplementary file 1 [file polymers-16-00039-s001.zip › Figure S1 Influence of aging time on filtration performance.tif]

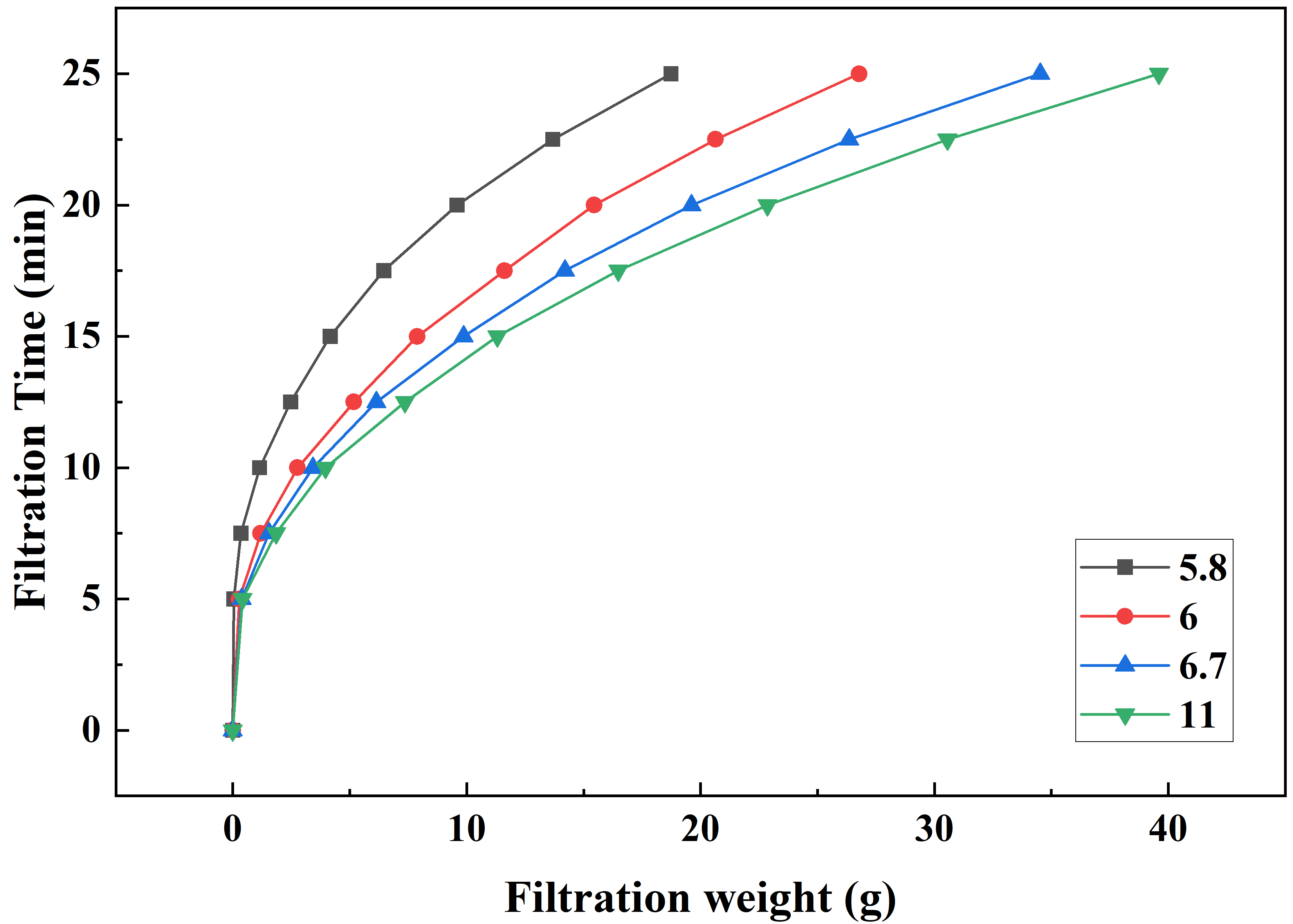

Supplement: Supplementary file 1 [file polymers-16-00039-s001.zip › Figure S2 Influence of stabilizer intrinsic viscosity on filtration performance.tif]

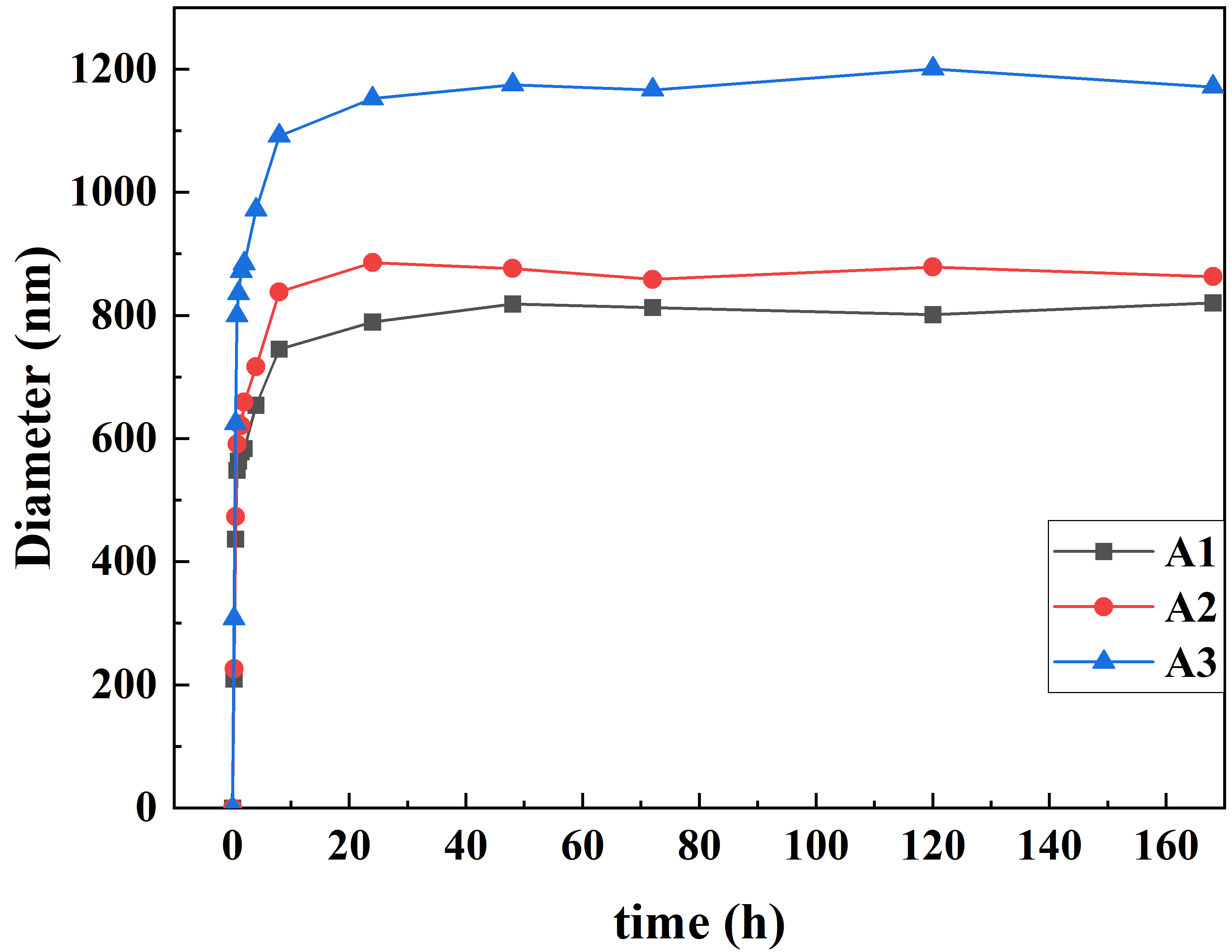

Supplement: Supplementary file 1 [file polymers-16-00039-s001.zip › Figure S3 Influence of stabilizer molecular weight (A1 A2 A3) in coils size on the reaction..tif]

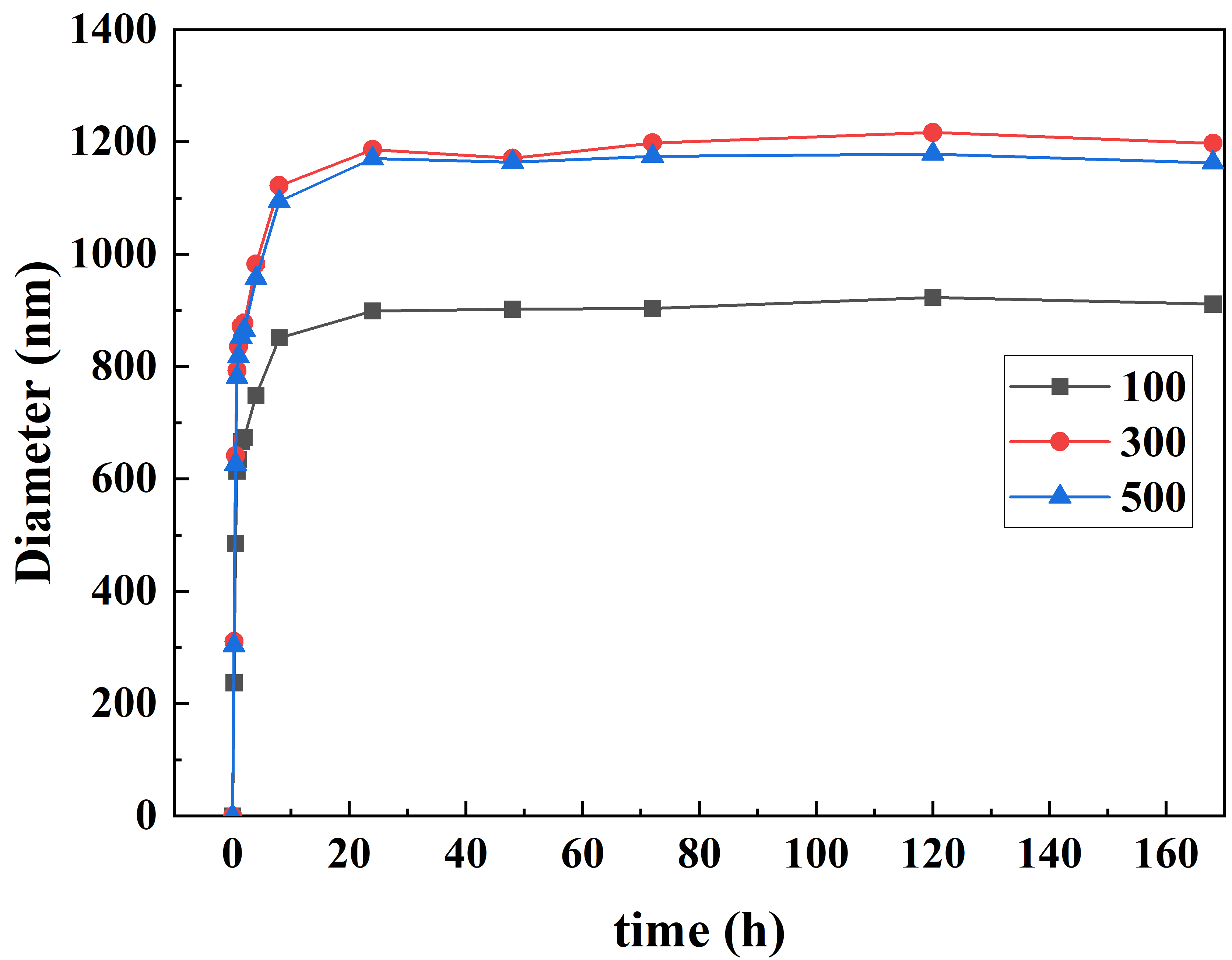

Supplement: Supplementary file 1 [file polymers-16-00039-s001.zip › Figure S4 Influence of stirring speed on coils size in the reaction.tif]
